# Pathway narratives towards a nature-positive European Union land system: operationalising the Nature Futures Framework for policy objectives

**DOI:** 10.1007/s11625-025-01737-0

**Published:** 2025-09-04

**Authors:** Joanna Raymond, Thomas M. Schmitt, Maximilian Tschol, Tristan R. M. Bakx, Lluís Brotons, Claire Brown, Robert Buitenwerf, Elizabeth Díaz-General, Anna Ferretto, Judith Kloibhofer, Tobias Laimer, Francisco Moreira, Sean E. H. Pang, Ella Plumanns-Pouton, Reinhard Prestele, Angus M. Smith, Jens-Christian Svenning, Mark Rounsevell

**Affiliations:** 1 Institute of Meteorology and Climate Research, Atmospheric Environmental Research (IMK-IFU), Karlsruhe Institute of Technology, Kreuzeckbahnstraße 19, 82467 Garmisch-Partenkirchen, Germany; 2 Institute of Geography and Geo-Ecology, Karlsruhe Institute of Technology, Kaiserstraße 12, Building 10.50, 76131 Karlsruhe, Germany; 3 School of Geosciences, University of Edinburgh, Drummond Street, Edinburgh, EH8 9XP UK; 4 CIBIO-BIOPOLIS, Campus de Vairão, 4485-661 Vairão, Portugal; 5 CIBIO, Centro de Investigação em Biodiversidade e Recursos Genéticos, InBIO Laboratório Associado, Instituto Superior de Agronomia, Universidade de Lisboa, Tapada da Ajuda, 1349-017 Lisbon, Portugal; 6 CSIC, 08193 Cerdanyola del Vallès, Spain; 7 CREAF, 08193 Cerdanyola del Vallès, Spain; 8 CTFC, 25280 Solsona, Spain; 9 Center for Ecological Dynamics in a Novel Biosphere (ECONOVO), Department of Biology, Aarhus University, Ny Munkegade 114, 8000 Aarhus C, Denmark; 10 Forest and Nature Conservation Policy Group, Wageningen University, P.O. Box 47, 6700 AC Wageningen, The Netherlands; 11 UNEP-WCMC, UN Environment Programme World Conservation Monitoring Centre, 219 Huntingdon Road, Cambridge, CB3 0DL UK

**Keywords:** European Green Deal, Policy objectives, Normative scenarios, Land system, Ecosystem services

## Abstract

The Nature Futures Framework (NFF) aims to open up space for plural perspectives on human–nature relationships and to identify visions for people to navigate towards desirable nature-positive futures. Operationalising the NFF offers a way to explore alternative pathways to reach these futures, but such applications are currently underdeveloped. Normative narratives can describe pathways towards nature-positive futures based on environmental policy objectives and the diverse values within and across societies. Here, we review the EU’s policy objectives for land systems, biodiversity, climate, and the environment, including the European Green Deal, to identify actions and create pathway narratives at the EU scale that lead to a nature-positive future. Specifically, we developed three pathway narratives along the three NFF value perspectives that are distinct in how they implement actions to achieve EU policy objectives. In Nature for Nature, the restoration of natural processes is prioritised, in Nature as Culture/One with Nature the connection of people with nature is emphasised, and in Nature for Society development focuses on safeguarding ecosystem services. Whilst clear differences in the valuation of nature between the pathway narratives may lead to trade-offs, we also see several potential synergies for meeting policy objectives. These narratives can inform models to quantify NFF-based scenarios to understand the trade-offs and synergies of achieving the EU’s policy objectives on land use, ecosystem provisioning, and quality of life. They can also serve as a basis for the creation of case study narratives within the EU through participatory processes.

**Supplementary Information:**

The online version contains supplementary material available at https://doi.org/10.1007/s11625-025-01737-0.

## Introduction

Earth’s biodiversity is rapidly deteriorating in response to human pressures, primarily due to land use change and the overexploitation of organisms (IPBES 2019). The widespread loss of species, habitats, and ecological processes not only threatens the existence of unique ecosystems, but also endangers the multitude of nature’s contributions to people (Díaz et al. 2019; IPBES 2019). Ecosystems are further threatened by the unprecedented changes in the Earth’s climate, a consequence of harmful human activities releasing greenhouse gases into the atmosphere (IPCC 2021).

To halt and reverse biodiversity loss and wide-scale ecosystem deterioration, urgent and collective action is required across all sectors of society (Díaz et al. 2019; Leclère et al. 2020; Mace et al. 2018; Runhaar et al. 2024). In this context, frameworks for sustainable transformation and transition are being proposed across regional, national, and international scales (Maron et al. 2021; Pattberg et al. 2019). For instance, the European Green Deal was launched in 2019 as a package of policy initiatives and legislative proposals that aimed to support the transition of the European Union (EU) to a “prosperous society that responds to the challenges posed by climate change and environmental degradation, improving the quality of life of current and future generations” (EU 2019, p. 23). Effectively delivering on these policy objectives requires a reorganisation of the land system that accounts for the diverse perspectives and values of people who use, manage, and benefit from land (Box 1; Chan et al. 2020; Dou et al. 2023; Meyfroidt et al. 2022). There is growing awareness that the land-based services that society chooses to value and prioritise will consequently shape the emerging synergies and trade-offs implicated in meeting multiple policy objectives (Howe et al. 2014; Meyfroidt et al. 2022). Exploring how to mitigate potential trade-offs and identify the synergies to deliver on policy objectives require narratives (see Box 1) that are normative (see Box 1) and target seeking. These narratives should describe desirable futures and the pathways to move towards them, based on the diverse values within and across societies (Arneth et al. 2019; Rosa et al. 2017).

To open up plural perspectives in the creation of nature-positive futures (see Box 1) that can be used to develop consistent scenarios and models, the Intergovernmental science–policy Platform on Biodiversity and Ecosystem Services (IPBES) developed the Nature Futures Framework (NFF, see Box 1) (Pereira et al. 2020). The NFF is a heuristic tool supporting the development of visions of desirable, nature-positive futures and pathways (i.e. the set of measures and actions that underpin the changes) to achieve these futures (see Box 1) (Kim et al. 2023; Pereira et al. 2020). The NFF seeks to consider the plurality in human–nature relationships (IPBES 2022) and distinguishes three primary value perspectives (see Box 1), termed “Nature for Nature” i.e. mainly drawing on nature’s intrinsic values and existence values; “Nature as Culture” (referring to Nature as Culture/One with Nature), i.e. mainly nature’s relational values and non-material benefits from nature; and “Nature for Society”, i.e. mainly a subset of nature’s instrumental values (see Box 1) (Kim et al. 2023; Pereira et al. 2020). Instrumental, intrinsic, and relational values cannot always be clearly separated and are sometimes held simultaneously by the same individuals (e.g. Arias-Arévalo et al. 2017; Himes and Muraca 2018; Schmitt et al. 2022). Thus, the NFF acknowledges that most people perceive themselves in intermediary positions of the value perspectives (Pereira et al. 2020).

To inform policymaking, quantitative models can be used to project the development of social–ecological systems into the future (IPBES 2016; Rounsevell et al. 2021). The implementation of the NFF value perspectives into these models requires narratives that guide the specification of various model parameters, rules, or elements for developing future scenarios (O’Neill et al. 2017). Durán et al. (2023) provide a set of imaginative storylines that are consistent with the NFF value perspectives and which cover a wide range of nature-related themes. However, these diverse themes are difficult to model with current, quantitative approaches, especially those approaches that address specific environmental components such as the land system. Relevant features and information within qualitative narratives that support translation into quantitative modelling exercises include qualitative descriptors and indicators that allow specification of where and over what time scale changes in the land system take place (Alcamo 2008; Booth et al. 2016; Mallampalli et al. 2016). Dou et al. (2023) present a first modelling study evaluating implementation pathways relating to the land system and the impacts of EU environmental policies (see Box 1) according to the NFF. Narratives that explore different implementation pathways can support policy design and agenda setting (IPBES 2016), but current NFF applications remain underdeveloped in terms of how well they embed policy objectives. Dou et al. (2023) focus on particular major policy objectives and a selected number of narrative elements that describe actions relating to no-net-loss of natural areas, the extension of protected areas, and the reduction of nitrogen use (Dou et al. 2023). Another recent study by D’Allesio et al. (2025) created a more holistic set of European land system narratives that put special attention on nature protection and the implementation of restoration measures within protected areas. However, there is still a need for additional NFF narratives that more comprehensively describe the multiple policy objectives of the EU relating to the land system. These narratives should not only go beyond narrow pathway explorations of individual modelling studies, but include a broad description of actions and measures underpinning the implementation of the European Green Deal and other land-related EU policies according to each NFF perspective.

In this context, this study aims to develop narratives that explore pathways at the EU level which strive towards achieving a desirable nature-positive future and can be used to inform policy design and implementation. We first present a nature-positive vision of the EU, based on a review of EU policies related to the land system, biodiversity, climate, and the environment. We then cluster these objectives into policy themes and for each theme, develop three pathway narratives for the land system. Each narrative follows a distinct NFF value perspective to achieve land-related EU policy objectives. In doing so, we identify multiple potential actions and measures for implementing the European Green Deal and other land-related EU policies across the EU, based on the diverse relationships between humans and nature in the land system.

Box 1: Glossary of terms **Nature Futures Framework:** A heuristic tool developed by the IPBES that captures diverse, positive human–nature relationships and the three values of nature: intrinsic, instrumental, and relational (Pereira et al. 2020).

**Intrinsic values:** The value of nature, ecosystems, or life as an ends in themselves, irrespective of their utility to humans (Arias-Arévalo et al. 2017).

**Instrumental values:** The value of an entity as merely a means to an end (Arias-Arévalo et al. 2017).

**Relational values:** The importance attributed to meaningful relations and responsibilities between humans and between humans and nature (Arias-Arévalo et al. 2017).

**Value perspectives**: Differing perspectives on the value of nature and on human–nature relationships. These underpin differing visions of and pathways towards nature-positive futures (Pereira et al. 2020).

**Scenarios:** A plausible description of the future that is based on a coherent and internally consistent set of assumptions about driving forces (e.g. rate of technological change) and relationships (IPCC 2023).

**Vision**: A desirable image of the future (Wiek and Iwaniec 2014).

**Normative:** Normativity refers to the existence of rules or standards that guide behaviours or actions based on beliefs or meanings (Glock 2015). Normative views, judgements, and choices in our work relate to the distinct value perspectives of the NFF.

**Narrative**: A narrative provides an imaginative and qualitative description of the future. It describes aspects that are difficult to project quantitatively (such as the quality of institutions, political stability, environmental awareness, etc.), and provides the logic underlying elements that are quantifiable (O’Neill et al. 2017).

**Pathway:** A pathway is a strategy for moving towards a future vision or target (Pereira et al. 2020).

**Policy instruments**: When referring to policy instruments, we have included both binding and non-binding instruments. Therefore, the resulting analysis uses all relevant strategies, regulations, directives, laws, and action plans.

**Policy objectives**: We use the word objectives to include all qualitative and quantitative targets, goals, pledges, commitments, and objectives set out within the relevant EU policy instruments identified in this study.

**Functioning ecosystem**: A functioning ecosystem supports the survival and reproduction of species and sustains ecological processes (such as nutrient cycling and energy flow) (Perino et al. 2019).

**Cultural landscapes**: Landscapes that are modified by human activity with intangible values and meanings. These include features that are threatened to change or disappear (Jones 1991; Tieskens et al. 2017).

**Land system**: The land system represents the terrestrial component of the Earth system. It encompasses all processes and activities related to the human use of land, including socioeconomic and technological conditions, the natural environment, the systems of governance, as well as the benefits gained from land and the unintended social and ecological outcomes of human activities on land (Verburg et al. 2013).

## Methods

Here, we present the methodological approach for creating normative pathway narratives for a nature-positive EU land system.

**Fig. 1:**
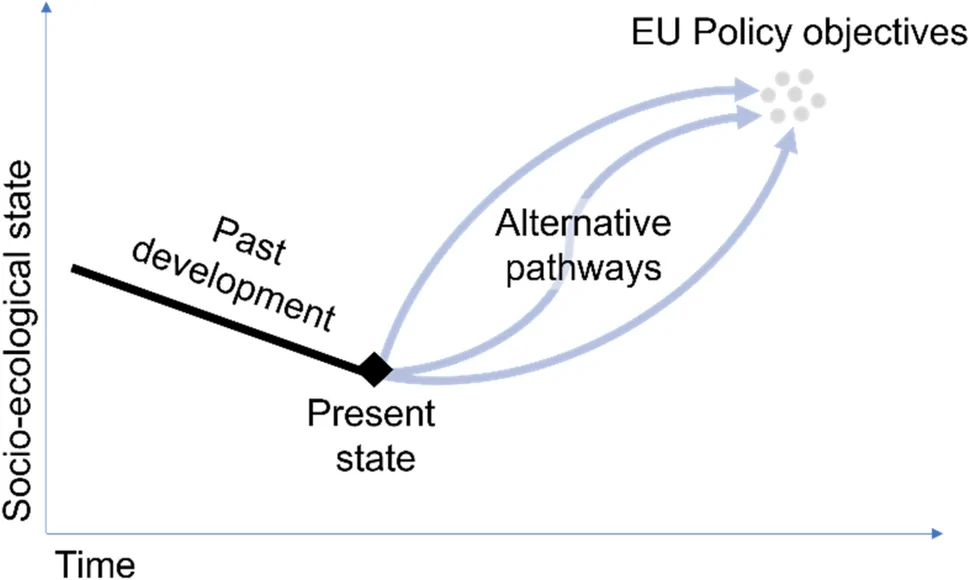
Conceptual overview of the study, linking past developments (black arrow) and the present state (black diamond) to a desirable future vision, targeting the EU policy objectives (grey dots). Pathways represent a set of nature-positive actions to achieve the EU policy objectives, based on the NFF value perspectives (blue arrows)

**Fig. 2:**
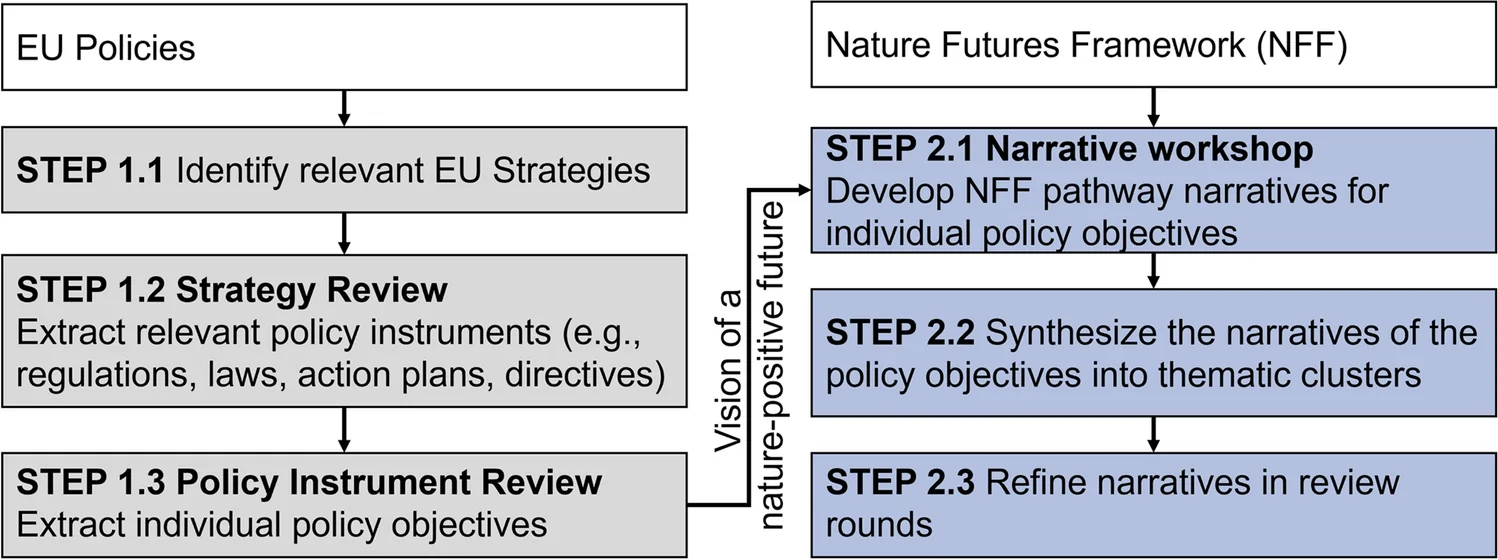
Overview of the procedure that drew on identified EU policies (Step 1) and the Nature Futures Framework (Step 2) (white boxes). EU policy objectives relating to the land system were identified and served as a vision of a nature-positive EU (grey boxes). NFF pathway narratives towards this vision were then developed (blue boxes)

We applied a target-seeking scenario approach by defining EU policy objectives as a vision for a desirable future EU land system (Step 1), and then developed narratives that outline alternative pathways to achieving this vision (Fig. 1). The pathways describe measures and actions that are based on the value perspectives of the NFF (Step 2). Steps 1 and 2 are summarised in Fig. 2.

**Step 1. Creating a vision of a nature-positive European Union based on existing policy objectives**

Within the context of the EU policy landscape, several instruments establish policy objectives that, if achieved, would contribute to a nature-positive EU. These instruments are developed and negotiated amongst a wide range of actors and vary in scope and level of ambition. To establish a vision for a nature-positive EU, we took a multi-step approach to identify and categorise relevant policy objectives in the EU context. We first (Step 1.1) determined key EU strategies that are relevant to land systems (see Table S1). This exercise was completed by searching the EUR-Lex online database run by the EU Publications Office (https://eur-lex.europa.eu). Specifically, we screened the European Green Deal and the EU Biodiversity Strategy to extract further strategies relevant to the land system. In determining relevance, we focused on strategies and policy objectives that directly addressed aspects of terrestrial land use, land cover, biodiversity conservation, and climate or environmental impacts associated with land management. Secondly (Step 1.2), each identified strategy was reviewed to determine the qualitative and quantitative objectives contained in its text, and to ascertain additional associated policy instruments relevant to the land sector (e.g. regulations, directives, and laws). We then reviewed the associated policy instruments to identify further qualitative and quantitative objectives contained in their text. Lastly (Step 1.3), all the policy objectives singled out in this exercise were then catalogued along with information on the status of the relevant policy instrument (e.g. legally binding versus non-binding, a proposed policy versus adopted policies that have come into effect) for the development of the pathways. By combining these policy objectives, we defined a vision of a nature-positive future EU land system (Fig. 1, grey dots).

**Step 2. Creating pathway narratives based on the Nature Futures Framework value perspectives**

In Step 2, we developed comprehensive narratives that outline alternative pathways to reaching the EU policy targets identified in Step 1. Firstly, we conducted a two-day, in-person workshop (Step 2.1). The workshop participants (*n* = 9) had an expertise in fields such as ecology (*n* = 7), social ecology (*n* = 5), geography (*n* = 3), political science (*n* = 2), nature conservation (*n* = 8), and land use change (*n* = 9). Participants come from a variety of different cultural backgrounds and nationalities, and have spent significant amount of time in various European countries. All participants in the workshop had already published research or are involved in ongoing projects working with the NFF.

The concepts used for the narrative development are based on the methodological guidance on the NFF (IPBES 2023). Descriptive characteristics of the NFF value perspectives, as described in Kim et al. (2023), served as a foundation for the workshop discussions. The workshop began with the development of a shared understanding of the NFF. Participants agreed to exclude indirect drivers of land use change beyond technological upscaling, and to not consider displacement effects of land use change outside of the EU at this stage. We ensured that the narratives contain the key components needed to represent actionable pathways towards sustainable futures, as outlined in Lembi et al. (2024). These included stating the broad vision and key features that will shape the scenario, choosing a clear set of values (as included in the NFF) and the location where the scenarios will take place throughout the narrative development process. While most expert-driven scenarios focus on quantitative model-based exercises, we applied a hybrid approach that is used in participatory scenario development including a combination of storylines and quantitative indicators (Biggs et al. 2015; Oteros-Rozas et al. 2015).

Following an initial exercise in which we outlined potential pathways for individual policy objectives, we created a set of three narratives for each policy objective, each reflecting a distinct NFF value perspective. Within each narrative, we described the pathway to reach a specific policy objective. This involved first identifying the required land use to reach the policy objectives and subsequently defining the associated management practices that may be conducted under each value perspective. The required land use and management practices were estimated by the authors, using their expertise and considering the information available in the policy documents. Participants engaged in a thorough discussion for each pathway narrative, aiming to ensure both coherence with the respective value perspective and plausibility. While certain policy objectives naturally align more closely with specific perspectives, our approach sought to explore how each objective could be realised under all three.

Next, we synthesised the resulting narratives for the individual policy objectives into a set of thematic clusters (Step 2.2). To achieve this, we started with an inductive approach to systematically categorise and group the pathway narratives for each of the individual policy objectives on thematic similarities. Through this iterative sorting process, we were able to distil the major elements from the individual narratives, which served as the foundation for constructing a set of synthesised narratives. Additionally, we created an overall narrative for the EU based on the narratives of all thematic clusters and extracted the key elements of each value perspective. Following the in-person workshop, we revised the resulting pathway narratives and included an additional group of external participants (*n* = 10) not involved in the previous workshop (Step 2.3). These reviewers assessed the narratives for consistency, internal logic, and alignment with the NFF value perspectives. All workshop participants (steps 2.1 and 2.2) and narrative reviewers (step 2.3) are authors of this paper or listed in the acknowledgements.

## Results

### A vision of a nature-positive European Union based on existing policy objectives

**Fig. 3:**
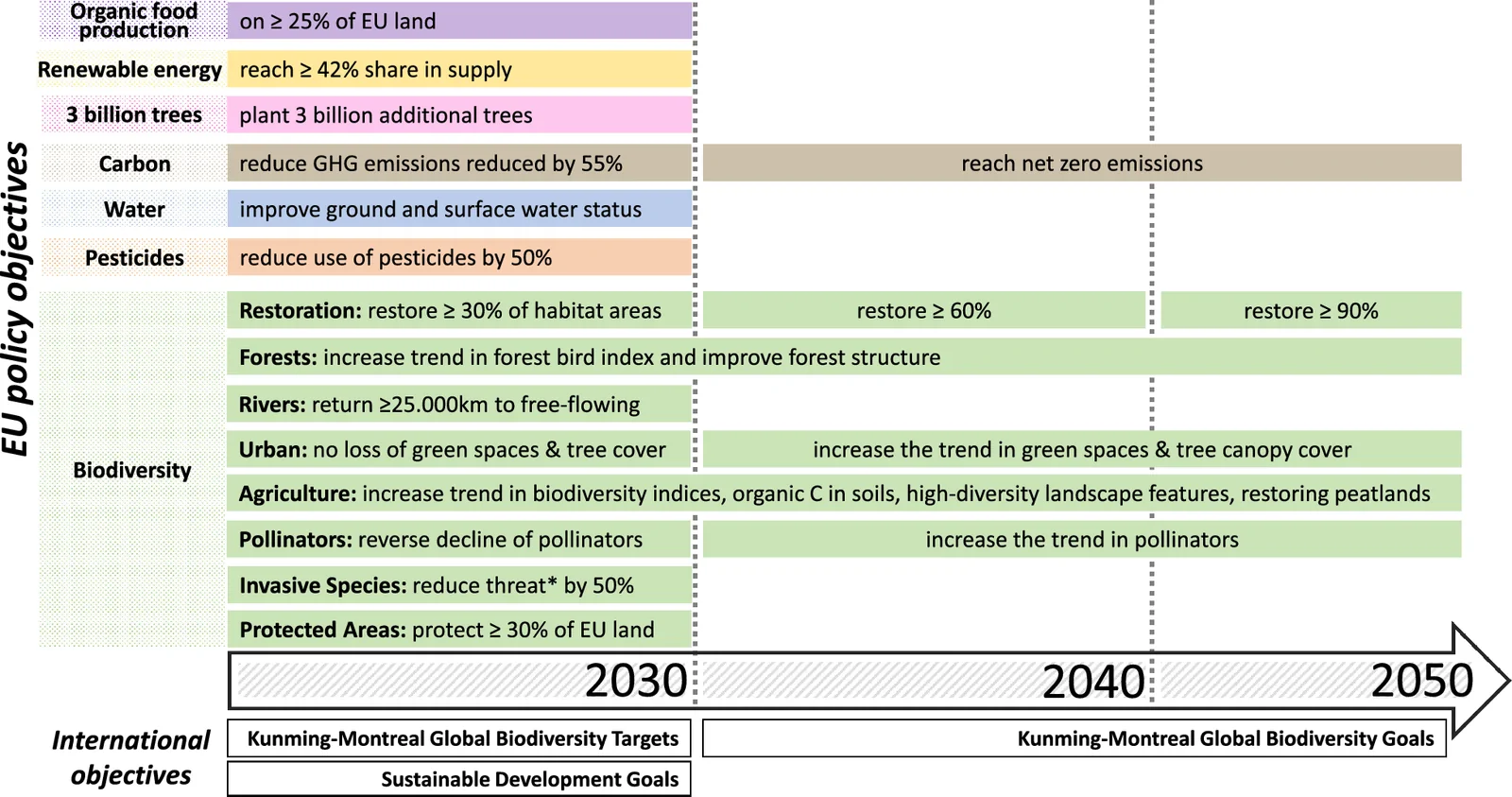
Overview of the timeline of identified European Union Policy objectives in their thematic clusters. *KM-GBF* Kunming–Montreal Global Biodiversity Framework, *SDG* sustainable development goals, *GHG* greenhouse gases. *Reduce the number of red List species threatened by invasive species. The seven major thematic clusters are shown in the first column

Based on the review of EU strategies and associated instruments, we identified several policy objectives. When combined, they constitute a democratically agreed vision of a desirable nature-positive future of the EU's land system. This vision includes ensuring sustainable food production, bringing nature back to agricultural land, expansion of biodiverse forests, climate mitigation, zero pollution, and restoration of various ecosystems. As the individual objectives were sometimes overlapping, we categorised the objectives into seven broader thematic clusters representing major policy areas relating to land use change (Fig. 3). For instance, the Farm to Fork strategy provides quantitative targets for several thematic clusters (organic food production, water quality and pesticides), whereby each cluster entails several more policy documents of a binding and non-binding nature. In the case of the pesticides cluster, this also includes the EU Action Plan “Towards Zero Pollution for Air, Water and Soil” and Directive 2009/128 “for Community action to achieve the sustainable use of pesticides” (a full list of the categorised policy objectives can be found in Table S1). The biodiversity category includes different sub-themes to capture the extent of the various objectives. Several of the EU objectives are linked to global frameworks, such as the 2030 Agenda for Sustainable Development and the Sustainable Development Goals (SDGs) (UN 2015), as well as multilateral environmental agreements (MEAs) and related implementation plans and other instruments such as the Kunming–Montreal Global Biodiversity Framework (KM-GBF) (CBD 2022) under the Convention on Biological Diversity (CBD). For example, the identified EU policy objectives on biodiversity link to SDG 17 Life on Land, and KM-GBF Targets 2 and 3, restoring 30% of degraded ecosystems and ensuring the conservation of at least 30% of the world’s land as protected areas by 2030, respectively.

### Pathway narratives to a nature-positive European Union

We present the overall EU narratives to a nature-positive EU land system in Table 1. In Nature for Nature, the restoration of natural processes is prioritised, through the strict protection of large land areas with a land sparing approach. In Nature as Culture, the connection of people with nature is emphasised, through land sharing and multifunctional landscapes, in which nature is integrated into culture and the surrounding landscapes. In Nature for Society, nature is balanced with the supply of ecosystem services for society through sustainable solutions in multifunctional landscapes (land sharing).

**Table 1:** A summary interpretation of the overall pathway narratives to a nature-positive European Union land system

Pathway narratives	Landscape approach	Land management
Nature for Nature	The EU prioritises the strict protection of large areas where ecological processes are restored with minimal human management, placing emphasis on land sparing approaches	With land being prioritised for nature, demand on land is met by land sparing approaches that sustainably intensify in high-yield areas that are of least importance for biodiversity, while organic farming uses agro-ecological and plant breeding solutions to increase yields where possible. Renewable energy and land use policies are tailored to reduce the ecological impact. Restoration practices with the most effective outcome for biodiversity are prioritised
Nature as Culture	The EU places emphasis on land sharing and multifunctional landscapes, which integrate nature into all aspects of local culture	Agriculture uses traditional, nature-positive land management practices leading to an emphasis on local solutions, as opposed to widespread technological interventions. Renewable energy and land use policies are tailored to maintain the integrity of cultural landscapes. Restoration practices focus on restoring native and culturally important species in cultural landscapes
Nature for Society	The EU balances the supply of ecosystem services for society with nature in multifunctional landscapes	Sustainable intensification of the most productive land utilises upscaled technology to increase the efficiency of inputs. Organic farming methods are used where possible to provide multiple ecosystem services. Renewable energy and land use policies incorporate technologies to increase the cost-effectiveness of integrating energy sources into multifunctional landscapes. Restoration practices focus on restoring the function of ecosystems to enhance the supply of ecosystem services

There are similarities and differences between the three perspectives, which are summarised in Fig. 3, for both the overall pathway narratives presented in Table 1 and the thematic clusters in Table S2. Upscaling of technology for sustainable intensification is a key feature of both Nature for Nature and Nature for Society, whilst land sharing and multifunctional landscapes are common in both Nature as Culture and Nature for Society. Employing renewable energy is a strategy in all of the perspectives, whilst strict protection to restore biodiversity, traditional practices in cultural landscapes (see Box 1), and cost-effectiveness and the provision of ecosystem services are distinct features in the narratives for Nature for Nature, Nature as Culture, and Nature for Society, respectively. Overlaps between Nature for Nature and Nature as Culture include the removal of river barriers to increase the length of free-flowing rivers and species protection in cultural landscapes.

The pathway narratives are synthesised in the following sections based on thematic clusters. For better comparison across value perspectives, we provide policy objective specific pathway narratives also in table format in the ESM (Table S2).

#### Nature for Nature pathway narrative

**Fig. 4:**
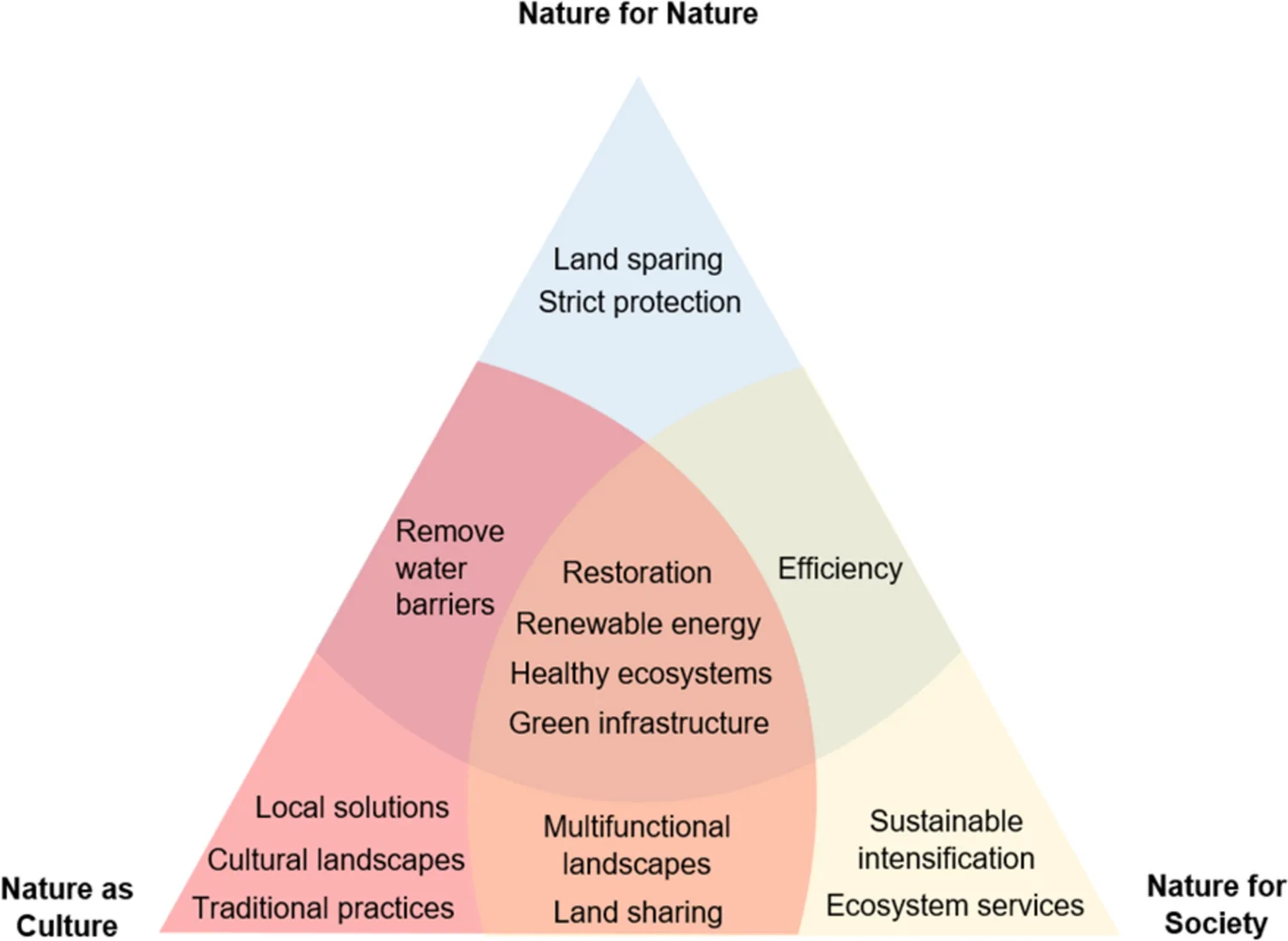
The key features within the pathway narratives. Features present in more than one value perspective are located in the overlapping regions of the triangle

In this nature-positive vision of the EU, land-related policy objectives are implemented such that decisions are made based on the intrinsic value of nature itself and to provide the best outcome for nature and biodiversity is prioritised. This involves both land management decisions and landscape planning that implement land sparing approaches. This is reflected in the selection of farmland for conversion to organic food production, such that farmland deemed most important for biodiversity, including high nature value farmland, is prioritised, whilst the upscaling and roll out of technology enable yield boosts in agro-ecological practices. In more intensive agricultural zones, precision technologies manage nitrogen fertiliser and pesticide use, reducing runoff. Water quality and quantity improve as water abstraction for irrigation is banned in ecologically sensitive areas, pesticides are eliminated from key biodiversity areas, and nitrogen fertiliser use is minimised to protect biodiversity (Fig. 4).

Tree planting to meet the additional three billion tree vision is arranged to provide the most ecological benefits. A climate-resilient mix of trees is established, with a preference for native species. These are planted or allowed to naturally regenerate on abandoned farmland, on previously intensively managed farmland or used in regenerating degraded forests, respecting ecological principles to support ecosystem functioning (see Box 1). These new and existing carbon sinks are protected, by avoiding deforestation of ecologically functional and old-growth forests. Efforts to increase carbon sinks focus on restoring heterogeneous forest landscapes, peatlands and other wetlands, to principally enhance biodiversity but also to contribute to emission reductions. The share of renewable energy increases, with larger wind and solar energy installations restricted to the least ecologically valuable locations and tailored to local conditions, with spatial arrangements that maintain connectivity between nature areas. Overall, emissions reductions from land use, land use change and forestry (LULUCF) are secondary to safeguarding biodiversity.

Restoration efforts to meet biodiversity targets create conditions for evolving, functioning ecosystems. This is enabled by restoring large, geodiverse single and cumulative natural and semi-natural areas with high trophic complexity, effective dispersal processes within and between them, and natural disturbance regimes.

Each ecosystem type is restored in such a way that it provides the most benefit to ecological processes and nature’s diversity. Within forests, a diverse, native, or ecologically equivalent mix of species is promoted in standing trees, deadwood, and open and semi-open areas within woodlands. Ecologically functional ecosystems are not used extractively, while outside of these areas, limited extractive use supports climate resilience through transitionary management and ecosystem restructuring. In urban areas, vegetated areas take priority over new developments, allowing a multi-species habitat mosaic to flourish. This includes tree health and abundance, as well as spontaneous vegetation. Living walls and rooftop gardens are created and valued for the increase in biodiversity they bring to urban areas. Connections to natural habitats are developed via nature corridors, where native flora and fauna are introduced and managed according to biodiversity needs. Farms are small- scale, use organic production methods, and are spatially arranged to promote connectivity and reduce ecosystem fragmentation, supporting diverse bird species, increasing pollinator populations and wildlife movement. Agricultural ecosystems transform through the use of sustainable farming practices and enhance the amount of semi-natural habitat within agricultural mosaics, improving diverse habitats that boost the grassland butterfly index and the farmlands bird index. Pastures and rangelands transition to trophic rewilding areas with free-roaming herbivores, preventing homogeneous succession to shrubland or forest and promoting mosaic vegetation. Rivers flow freely, as anthropogenic barriers such as dams, weirs, and locks are removed, allowing the return of natural hydrological dynamics, reconnecting rivers to floodplains, and facilitating the return of ecosystem engineers such as beavers and water voles.

Minimal human intervention in important ecosystems is of high priority, and all of the EU’s protected areas (PAs) are under strict protection as ‘strict nature reserves’ (IUCN category *Ia*) or ‘wilderness areas’ (*Ib*). PA coverage includes the most ecologically valuable areas, including old-growth forests. The PA network grows through restoration efforts and expansion of natural corridors. A small proportion of PAs allow for limited and sustainable resource use, such as when subsistence resource use of local communities contributes to conserving a specific ecosystem where species have become dependent on human-modified landscapes, e.g. alpine meadows or traditional orchards.

#### Nature as Culture pathway narrative

The transition towards a nature-positive future EU, in which societies, cultures, and traditions are intertwined with nature, is reflected in the role of cultural landscapes in embedding sustainable practices as culture. Thus, land decisions are made based on the maintenance of social constructions that depend on nature, but which reciprocally respect and appreciate it, co-existing in a harmonious manner. Conventional farmlands deemed most culturally valuable are the first to transition to organic production. This ensures that organic food systems are well integrated into local society and culturally significant landscapes. Efforts to reduce fertiliser usage outside of organic areas use minimal and targeted application, drawing on traditional fertilisation and manure practices. Pesticide usage also decreases in cultural landscapes, especially where they threaten culturally important species, such as bees for beekeeping. To enable water bodies to recover from a history of harmful practices, water body restoration projects are designed in harmony with cultural activities, to honour people’s non-material connections to water.

There is significant community engagement in tree planting efforts to meet the three billion tree vision. Local communities plant climate-resilient and culturally significant tree species, both on agricultural land to expand traditional agroforestry systems, as well as in other accessible areas. Decisions about where to plant trees are made locally. In regions where open or semi-open landscapes are culturally valued or ecologically important, tree planting is more limited to respect local perceptions and ecological characteristics. High accessibility and involvement in the planting process encourage communities to also engage in protecting reforested areas.

The protection and careful management of young and mature forest also contribute to increased carbon sinks and reductions of greenhouse gas emissions from the land sector. Deforestation is avoided, whilst nature-based solutions, such as selective logging for long-lived wood products and construction, are promoted, provided they do not contradict local traditions and ecosystems. Peatland and wetland restoration projects balance cultural benefits with emission reductions. Extensive forms of agriculture (e.g. paludiculture) are allowed on restored peatlands and other wetlands, as well as rewetting peatlands, allowing experiences and activities that foster human–nature interactions. Reductions in emissions from the energy sector come as society embraces renewables, including bioenergy sources. Multifunctional landscapes promote the integration of renewable energy into local culture, such as through the use of small-scale agri-voltaics and widespread use of existing buildings for placement of photovoltaics. Utility-scale energy production sites are located outside of landscapes valuable for nature and culture. Overall however, reducing emissions in the land sector is secondary to maintaining cultural landscapes, with faster emission reductions in other sectors limiting the need for a compensating LULUCF sink.

Ecosystem restoration activities provide a range of co-benefits for nature and culture. This is enabled through the expansion of traditional management practices that maintain cultural landscapes, through prioritising habitat restoration and connectivity for species in traditionally important landscapes, such as alpine grasslands, and through avoiding the loss of traditional land use practices and landscape features such as hedgerows and ponds.

Each ecosystem is restored in a culturally appropriate way, with the participation of local communities. In river systems, local communities participate in watershed management and remove river barriers in landscapes that allow for cultural practices, fostering a spiritual connection with nature. In urban areas, the restoration of green spaces enhances the connections between people and nature through active involvement and through increasing access to nature. This occurs in multiple ways, through the greening of gardens using local species, by creating community gardens to provide opportunities to share traditional sustainable farming practices in an urban setting, and by converting disused building and brownfield sites to both open greenspaces and forested areas. New green spaces are also created through initiatives such as living walls and rooftop gardens, and facilitate greater everyday interaction with nature in urban areas. In forests, both within and outside urban areas, extraction is permitted for construction of sustainable housing using mixed-age timber to increase the unevenness of the forest age and to allow for the build-up of deadwood and organic carbon. Small forest patches and other woody features are protected as landscape features in agricultural ecosystems, which helps to increase the share of agricultural land with high-diversity landscape features. Combined with careful planning and management by local communities and farmers, this contributes to the increase in the overall farmland bird index and bird populations in high nature value farmlands, through emphasising the protection of birds with the greatest cultural and spiritual importance. Extensive grazing of grassland habitats improves the grassland butterfly index. Increase in culturally significant pollinator species, such as bees for beekeeping, comes from an increase in pollinator-friendly plant species and the reduction of pesticides.

The protection of ecosystems takes place to provide both cultural and biodiversity benefits, resulting in both an increase in privately and publicly protected areas. The majority of the EU’s PAs are designated as ‘protected landscape’ (*V)* located in cultural landscapes and mainly co-managed with local communities, with the aim to protect biodiversity and conserve societies’ relational values of people with nature. Strict protection of PAs as ‘strict nature reserve’ (*Ia)*, ‘wilderness areas’ (*Ib),* and ‘national park’ *(II)* are preferentially located in areas that do not require the conversion of human-shaped, culturally important landscapes. Stricter protection of specific local sites as ‘natural monument or feature’ (*III)* occurs throughout cultural landscapes, to protect outstanding natural features with their associated biodiversity and to conserve spiritual and cultural values of such sites. To build a PA network, additional sites are protected throughout and between cultural landscapes ‘habitat or species management areas’ (*IV)*, with the aim to provide managed nature corridors for key species.

#### Nature for Society pathway narrative

Through the implementation of the EU policy objectives, the land system is transformed to provide a multitude of benefits for nature and society. This is exemplified through the conversion of farmland to organic production, such that areas are selected where it improves the supply of ecosystem services, such as groundwater supply, whilst avoiding high-productivity areas to prevent conflicts with food security. Use of technologies such as precision agricultural and vertical farming enable more targeted and moderated usage of pesticides and fertilisers, thus improving water quality while preventing yield losses. Agroforestry systems are implemented to provide multiple ecosystem services, including reducing wildfire risk. The societal benefits of planting three billion additional trees are increased by the location of climate-resilient tree species, such as in urban heat islands to provide shading, and by selecting the best trees for climate change mitigation for use in offsetting in carbon markets.

Greenhouse gas emission reductions from the land sector are achieved by promoting a variety of nature-based solutions that benefit both biodiversity and people. Carbon sinks increase by avoiding deforestation, managing forests through selective logging for long-lived wood products, and planting tree species that provide long-term climate benefits. Peatlands and other wetlands are restored, allowing for paludiculture and rewetting for recreational purposes that foster economic benefits for local communities. Society meets its energy needs through the expansion of renewable energy sources, with high-intensity solar and wind farms used in areas less important for other ecosystem services, while multifunctional solutions such as agri-voltaics are employed where there are additional benefits for society. Bioenergy production focuses on small-scale second-generation energy crops and residual forest products.

Ecosystem restoration also focuses on providing multiple ecosystem services, including carbon storage and risk reduction to extreme events, and there is an emphasis on climate-smart solutions. This includes sustainable forest management that promotes standing deadwood, increases forest age diversity and soil organic carbon, increasing forest resilience. Restored water bodies and wetlands provide provisioning and regulating ecosystem services, such as additional recreational access, through increasing the meanders in rivers, and flood risk reduction, through river dredging. Urban areas are further protected by flooding meadows downstream, to create natural retention basins. Green spaces take a diversity of forms within urban areas, such as through the incorporation of living walls into buildings and rooftops, allowing for both building developments and increased green space, as well as urban gardens, which contribute to local food security. Food security is further enhanced by the restoration of agricultural ecosystems that contribute to food production, such as by reintroducing small landscape features in intensively farmed land and increasing organic carbon in cropland mineral soils. The farmland bird index rises due to the protection of birds that provide benefits to society, e.g. for pest control and recreational activities. Pollinator diversity increases through improved pesticide management and floral and nesting resources, to provide a pollination service to farmland.

To enable the supply of provisioning and regulating ecosystem services, nature within the EU is safeguarded as ‘protected area with sustainable use of natural resources’ (*VI*). Strict protection only takes place where the benefits of regulating services outweigh the costs of prohibited resource use, such as in ‘national parks’ (*II*) where tourism and recreation is still permitted. Stricter protection of specific local sites as ‘natural monuments or features’ (*III*) occurs throughout multifunctional landscapes to protect outstanding natural features and their associated biodiversity for tourism and recreation. To build a network of protected areas, additional sites are safeguarded as ‘habitat or species management areas’ (*IV*), creating managed nature corridors for key species for ecosystem functioning and society.

## Discussion

Conceptualising a nature-positive EU through the NFF value perspectives resulted in three distinct pathways for implementing EU policy objectives for the land system. These differences arose due to their distinctive value perspectives of nature and the subsequent configuration of land management strategies.

### Commonalities and distinctions between pathway narratives in implementing policy objectives

As intended, all three pathway narratives share a common commitment to biodiversity conservation, but represent key characteristics of each value perspective, such as cultural landscapes in Nature as Culture, the maintenance of ecosystem services in Nature for Society, and strict nature protection in Nature for Nature (Fig. 2), similar to narratives developed in previous studies (e.g. d'Allesio et al. 2025; Durán et al. 2023; Kim et al. 2023). Despite such characteristic differences, some narrative elements occur in multiple pathway narratives. For instance, land sharing explicitly occurs in both the Nature as Culture and the Nature for Society pathways, which is similar to the narratives of Durán et al. (2023) and D’Allesio et al. (2025), whilst the removal of water barriers occurs in both the Nature for Nature and Nature as Culture pathway narratives. Some form of sustainable intensification in agriculture features in the Nature for Society and the Nature for Nature pathway narratives presented here and in Durán et al. (2023), while D’Allesio et al. (2025) do not mention solutions to sustainable intensification, but focus on separating conservation areas from high-intensity agricultural areas and its associated negative impacts on biodiversity.

The pathway narratives also share some of the identified synergies to reach multiple policy objectives within a pathway narrative. For instance, to meet the renewable energy objective, renewable energy can be integrated into multifunctional landscapes, such as through agri-voltaics, supporting both energy needs and cultural heritage that are valued in both the Nature for Society and Nature as Culture perspectives. Likewise, increasing the share of renewable energy usage is also important for reaching the carbon objectives across all three pathway narratives. The upscaling of technology and use of novel practices to increase agricultural yield could save land area required for food and reduce fertiliser inputs, which would result in benefits for biodiversity and society, valued in the Nature for Nature and Nature for Society perspectives. Further, the restoration of urban green spaces would enhance biodiversity, support human well-being (Reyes-Riveros et al. 2021), and promote ecosystem services, such as air purification, carbon storage, and cooling (Derkzen et al. 2015), which are valued in the Nature as Culture and Nature for Society perspectives. Across all pathway narratives, the aim to increase the share of organic production aligns with reducing pesticide and fertiliser use and should contribute to improvements in water quality through reduction in leakage effects.

While we translated EU policy objectives into a combined vision that all three pathway narratives aim to achieve, we acknowledge some limitations arising from this approach. First, the formulation of some policy objectives inherently aligns better with certain value perspectives. In particular, currently agreed policy objectives predominantly express a Nature for Society perspective. For example, the various approaches for achieving the carbon objectives are directly linked to the ecosystem service for carbon sequestration, aligning well with the Nature for Society narrative. Meanwhile, the policy objective of increasing organic food production to 25% implies a greater land area for low intensity farming (Tscharntke et al. 2021), contradicting the land sparing approach within the Nature for Nature narrative. Instead of focusing on the area under organic farming, which can still have negative impacts for biodiversity (Tscharntke et al. 2021), an alternative policy objective that is more aligned with Nature for Nature may thus require land sparing to be implemented within a mosaic landscape including higher yielding intensive agricultural areas. Secondly, extent of trade-offs in achieving biodiversity goals may likely differ across the three NFF perspectives, with the Nature for Nature pathway offering the broadest and least compromised set of solutions, whereas Nature as Culture and Nature for Society must navigate trade-offs with cultural values and societal demands, respectively. Third, the developed vision only represents a subset of the EU policy landscape and the identified objectives are strongly interrelated with other sectors not included in the study. For example, meeting carbon targets also requires a transformation of the transport and manufacturing sectors, given that, in 2020, the contribution of these sectors to greenhouse gas emissions was more than 2.6 times that of agriculture (Climate Watch 2024).

### Changes in indirect drivers of land use

Our narratives do not assume significant changes in governance, technology, economic and other societal aspects that may affect land use change. We chose not to impose such changes on the pathway narratives, but provide the opportunity for changes in those indirect drivers to be combined with the narratives in future studies.

There is significant opportunity to reduce the demand on land through societal changes in dietary choices, reduction in energy consumption, and other lifestyle changes. There is a large body of evidence indicating that widespread dietary shifts from animal-based food to plant-based foods could reduce greenhouse gas emissions from direct agricultural production (e.g. Hentschl et al. 2023; Scarborough et al. 2023; Sun et al. 2022) and, by freeing up land previously used for agriculture (Alexander et al. 2016), create space for more restoration, increasing carbon storage and providing biodiversity benefits (Eisen and Brown 2022; Kozicka et al. 2023). Reducing food waste would further amplify this effect (Read et al. 2022). Similarly, changes in lifestyle that reduce energy and resource consumption, such as through incorporation of more active transport, reducing household energy usage through improved insulation, and a transition to a circular economy, can further reduce demands on land, whilst also reducing carbon emissions and pollution (Cantzler et al. 2020; Gallego-Schmid et al. 2020; Mishra et al. 2014; Moriarty and Honnery 2019). Importantly, demand-level changes will likely align differently with each pathway narrative. For example, in the Nature for Nature narrative, anthropogenic production may be modified to align with the limits of nature, requiring much more drastic shifts in consumption, whilst in the Nature as Culture and Nature for Society narratives, dietary changes to benefit nature may be balanced with cultural and societal demands.

Governance represents another key indirect driver of land use change. Impactful shifts at the policy level—such as ambitious reforms of the European Union’s Common Agricultural Policy (CAP)—could play a pivotal role. For instance, redirecting funds from area-based direct payments with low environmental requirements to effective agri-environmental and climate measures and eco-schemes would help operationalise nature-positive strategies (Pe’er et al. 2020). Given that the CAP seeks to balance a broad range of social, environmental, and economic goals, implementing NFF-aligned versions of CAP reforms could serve as a lever for realising the land-based measures and actions proposed in our study. As an indirect driver, such governance reform can shape both the incentives and constraints that influence land use decisions on the ground.

Technological changes could also affect land use via transformative innovations or paradigm shifts, such as speculative or breakthrough technologies like nuclear fusion. However, especially given the limited time horizon of policy objectives, we chose to limit our narratives to the upscaling of existing practices, tools, and approaches that are already accessible and proven, thereby trying to maintain consistency with the decision not to impose significant changes in indirect drivers. This keeps the narrative flexible enough to be used by follow-up studies without being too prescriptive.

### Potential use of the policy pathway narratives in modelling and co-creation processes

The narratives presented here can support the parametrisation of social–ecological models that seek to simulate future environmental impacts, quantitatively assess synergies and trade-offs in policy objectives, and rely on land system trajectories across plural perspectives of human–nature relationships. Methods for translating qualitative narratives into quantitative modelling exercises rely on linguistic expressions that support quantification (Alcamo 2008; Kok et al. 2015; Mallampalli et al. 2016). This can include the expression of trends in terms of quantifiable units (e.g. per cent increase), expressions that highlight relative changes without assigning quantities (“more”, “less”), as well as guiding principles for spatial allocation decisions (“placed outside”, ”restricted to”). The description of policy objectives presented in this study supports parameterisation by specifying the temporal trajectory and area-based information over which changes should occur, while the narratives themselves are rich in qualitative descriptors that allow interpretation of the relevant land use and land cover types, as well as the kind of actions and measures to bring about changes in indicators or biophysical properties of the land system. For instance, while the policy objective on renewable energy specifies a quantifiable trend towards an increased share of renewable energy in 2030, the associated narrative elements provide information on the favoured type of renewable energy installation, as well as normative reasoning guiding their spatial allocation. For modelling applications, the narratives can be combined with assumptions on indirect drivers of land use and biodiversity loss. These indirect drivers could be derived in multiple ways, including projections of drivers from existing databases, such as the OECD–FAO Agricultural Outlook (OECD and FAO 2021) and the OECD long-term baseline projections for indicators (OECD 2024), driver descriptions from the Shared-Socioeconomic Pathways (SSPs) (e.g. Alexander et al. 2023; Dou et al. 2023), or stakeholder- or expert-derived narratives on drivers (Harmáčková et al. 2022).

The narratives can also inform the creation of policy implementation pathways using stakeholder engagement or co-creation approaches. For example, stakeholder-specific, policy-based pathways can provide additional qualitative features relevant to local contexts which are not depicted in the pathway narratives presented here, while still relying on the policy objectives identified here. We have also assumed that the objectives set out in EU policy constitute a shared vision of a nature-positive EU within the current socially agreed political, governing, and institutional context. However, this will likely not be the case when the policymaking process is shaped by power imbalances of different stakeholders, potentially reducing the usefulness or legitimacy of pathway narratives that intend to achieve a pre-set vision of the future (Lemos et al. 2018; Turnhout et al. 2020). Models should thus aim at increasingly incorporating a plurality of NFF narratives of pathways and/or visions developed by different people to promote democracy in quantitative modelling, and to account for the uneven power dynamics within the EU and the resulting unequal distribution of land-based benefits (Meyfroidt et al. 2022). It is common practice in scenario-development processes to explore outcomes and check for plausibility of narratives with stakeholders (Biggs et al. 2015). This may then feed back into revised context-specific narratives.

The pathway narratives are situated within, and constrained by, the existing EU and international policy and legal landscape, which provides both opportunities and limitations for their implementation. While we considered policy objectives from both binding and non-binding instruments, the narratives assume continuity in current governance structures and exclude transformative societal, technological, or economic shifts. Many objectives align with major international agreements such as the Paris Agreement and the Kunming–Montreal Global Biodiversity Framework, though the narratives vary in their degree of alignment based on value orientations. Importantly, while binding instruments like the European Climate Law and Nature Restoration Regulation offer legal certainty, their effectiveness depends on clear definitions—such as what constitutes a “good condition” ecosystem—and on the development of meaningful, ecosystem-specific indicators.

## Conclusion

Conceptualising pathways towards a nature-positive EU through the lens of the three perspectives outlined in the Nature Futures Framework has led to the identification of three distinct pathways for implementing EU policy objectives related to the land system. These pathways, while unified in their commitment to biodiversity conservation and sustainable resource use, reflect different value perspectives on nature, and relationships between people and biodiversity, resulting in diverse land management strategies. They therefore provide a foundation for supporting quantitative modelling, and stakeholder engagement, all of which are crucial for opening the space for inclusive, democratic policy implementation decisions aligned with both EU and international environmental commitments.

## Supplementary Information

Below is the link to the electronic supplementary material.

Supplementary file1 (DOCX 59 KB)

## Data Availability

All data supporting the findings of this study are available within the paper and its supplementary information.
