# Supplementary material for: Pathway narratives towards a nature-positive European Union land system: operationalising the Nature Futures Framework for policy objectives
Source: Sustain Sci. 2025 Sep 4;21(5):2041–55. doi: 10.1007/s11625-025-01737-0 (PMC13680311; doi:10.1007/s11625-025-01737-0)
Supplement: Supplementary file 1 [file 11625_2025_1737_MOESM1_ESM.docx]

**Electronic Supplementary material (ESM)**

Table S1: Table summarising the instruments and specific policy objectives included within the thematic clusters. Objectives that indicate a continuation beyond e.g. 2030 but do not indicate a cut-off date are shown as e.g. 2030+.

| **Thematic cluster** | **Instruments** | **Binding/**  **Non-Binding** | **Policy objectives (policy commitments, goals and targets included in the text)** | **Timeline to achieve the policy objective** |
| --- | --- | --- | --- | --- |
| Organic food production | Communication from the Commission to the European Parliament, the Council, the European Economic and Social Committee and the Committee of the Regions  A Farm to Fork Strategy for a fair, healthy and environmentally-friendly food system.  COM/2020/381 final  <https://eur-lex.europa.eu/legal-content/EN/TXT/?uri=celex%3A52020DC0381> | Non-binding | *2.1 Ensuring sustainable food production*  This approach will help to reach the objective of at least 25% of the EU’s agricultural land under organic farming by 2030 and a significant increase in organic aquaculture. | 2030 |
|  | Communication from the commission to the european parliament, the council, the european economic and social committee and the committee of the regions  EU Biodiversity Strategy for 2030 Bringing nature back into our lives  COM(2020) 380 final  <https://eur-lex.europa.eu/legal-content/EN/TXT/HTML/?uri=CELEX:52020DC0380> | Non-binding | *2.2.2 Bringing nature back to agricultural land*  Organic farming in particular holds great potential for farmers and consumers alike. …To make the most of this potential, at least 25% of the EU’s agricultural land must be organically farmed by 2030. | 2030 |
| Renewable energy | Directive (EU) 2018/2001 of the European Parliament and of the Council of 11 December 2018 on the promotion of the use of energy from renewable sources  (Version 16/07/2024)  <http://data.europa.eu/eli/dir/2018/2001/2024-07-16> | Binding as to the result to be achieved (Article 288 TFEU)g | *Article 3. Binding overall Union target for 2030*  1.Member States shall collectively ensure that the share of energy from renewable sources in the Union’s gross final consumption of energy in 2030 is at least 42,5 %. | 2030 |
| 3 billion additional trees | Communication from the commission to the european parliament, the council, the european economic and social committee and the committee of the regions  'Fit for 55': delivering the EU's 2030 Climate Target on the way to climate neutrality  COM(2021) 550 final  <https://eur-lex.europa.eu/legal-content/EN/TXT/?uri=CELEX%3A52021DC0572> | Non-binding | *3.3.Re- and afforestation of biodiverse forests*  The EU Biodiversity Strategy for 2030 sets out a pledge to plant at least 3 billion additional trees by 2030 in full respect of ecological principles. This initiative will act against the ongoing trend of a declining net increase of the EU forest area. Over time, it will also contribute to increasing the EU forest cover and, with that, the EU land carbon sink and stock. It will also help raise societal awareness and commitment, contributing to reaching the objective of becoming the first climate neutral continent by 2050, to biodiversity restoration and to the circular economy. | 2030 |
|  | Communication from the commission to the european parliament, the council, the european economic and social committee and the committee of the regions  EU Biodiversity Strategy for 2030 Bringing nature back into our lives  COM(2020) 380 final  https://eur-lex.europa.eu/legal-content/EN/TXT/HTML/?uri=CELEX:52020DC0380 | Non-binding | *2.2.4.Increasing the quantity of forests and improving their health and resilience*  To make this happen, the Commission will propose a dedicated EU Forest Strategy in 2021 in line with our wider biodiversity and climate neutrality ambitions. It will include a roadmap for planting at least 3 billion additional trees in the EU by 2030, in full respect of ecological principles. | 2030 |
|  | Regulation (EU) 2024/1991 of the European Parliament and of the Council of 24 June 2024 on nature restoration and amending Regulation (EU) 2022/869  [*http://data.europa.eu/eli/reg/2024/1991/oj*](http://data.europa.eu/eli/reg/2024/1991/oj) | Binding | *Article 13 Planting three billion additional trees*  1. When identifying and implementing the restoration measures to fulfil the objectives and obligations set out in Articles 4 and 8 to 12, Member States shall aim to contribute to the commitment of planting at least three billion additional trees by 2030 at Union level  2. Member States shall ensure that their contribution to fulfilling the commitment set out in paragraph 1 is achieved in full respect of ecological principles, including by ensuring species diversity and age-structure diversity, prioritising native tree species except for, in very specific cases and conditions, non-native species adapted to the local soil, climatic and ecological context and habitat conditions that play a role in fostering increased resilience to climate change. The measures to achieve that commitment shall aim to increase ecological connectivity and be based on sustainable afforestation, reforestation and tree planting and the increase of urban green space. | 2030 |
| Carbon | Regulation (EU) 2021/1119 of the European Parliament and of the Council of 30 June 2021 establishing the framework for achieving climate neutrality and amending Regulations (EC) No 401/2009 and (EU) 2018/1999 (‘European Climate Law’)  <http://data.europa.eu/eli/reg/2021/1119/oj> | Binding | *Article 4 Intermediate Union Climate targets*  1. In order to reach the climate-neutrality objective set out in Article 2(1), the binding Union 2030 climate target shall be a domestic reduction of net greenhouse gas emissions (emissions after deduction of removals) by at least 55 % compared to 1990 levels by 2030. | 2030 |
|  | Regulation (EU) 2021/1119 of the European Parliament and of the Council of 30 June 2021 establishing the framework for achieving climate neutrality and amending Regulations (EC) No 401/2009 and (EU) 2018/1999 (‘European Climate Law’)  <http://data.europa.eu/eli/reg/2021/1119/oj> | Binding | *Article 2 Climate-neutrality objective*  1.Union-wide greenhouse gas emissions and removals regulated in Union law shall be balanced within the Union at the latest by 2050, thus reducing emissions to net zero by that date, and the Union shall aim to achieve negative emissions thereafter. | 2050 |
| Water quality and quantity | Directive 2000/60/EC of the European Parliament and of the Council of 23 October 2000 establishing a framework for Community action in the field of water policy  (Version 20/11/2014)  <http://data.europa.eu/eli/dir/2000/60/2014-11-20> | Binding as to the result to be achieved (Article 288 TFEU) | *Article 4 Environmental objectives*  1. In making operational the programmes of measures specified in  the river basin management plans:  (a) for surfacewater  (i) Member States shall implement the necessary measures to prevent deterioration of the status of all bodies of surface water, subject to the application of paragraphs 6 and 7 and without prejudice to paragraph 8;  (ii) Member States shall protect, enhance and restore all bodies of surface water, subject to the application of subparagraph  (iii) for artificial and heavily modified bodies of water, with the aim of achieving good surface water status at the latest 15 years after the date of entry into force of this Directive,..  (iii) Member States shall protect and enhance all artificial and heavily modified bodies of water, with the aim of achieving good ecological potential and good surface water chemical status at the latest 15 years from the date of entry into force of this Directive, in accordance with the provisions laid down in Annex V, subject to the application of extensions determined in accordance with paragraph 4 and to the application of paragraphs 5, 6 and 7 without prejudice to paragraph 8;  (iv) Member States shall implement the necessary measures in accordance with Article 16(1) and (8), with the aim of progressively reducing pollution from priority substances and ceasing or phasing out emissions, discharges and losses of priority hazardous substances without prejudice to the relevant international agreements referred to in Article 1 for the parties concerned;  (b) for groundwater  (i) Member States shall implement the measures necessary to prevent or limit the input of pollutants into groundwater and to prevent the deterioration of the status of all bodies of groundwater, subject to the application of paragraphs 6 and 7 and without prejudice to paragraph 8 of this Article and subject to the application of Article 11(3)(j);  (ii) Member States shall protect, enhance and restore all bodies of groundwater, ensure a balance between abstraction and recharge of groundwater, with the aim of achieving good groundwater status at the latest 15 years after the date of entry into force of this Directive, in accordance with the provisions laid down in Annex V, subject to the application of extensions determined in accordance with paragraph 4 and to the application of paragraphs 5, 6 and 7 without prejudice to paragraph 8 of this Article and subject to the application of Article 11(3)(j);  (iii) Member States shall implement the measures necessary to reverse any significant and sustained upward trend in the concentration of any pollutant resulting from the impact of human activity in order progressively to reduce pollution of groundwater. | 2015 |
|  | Communication from the Commission to the European Parliament, the Council, the European Economic and Social Committee and the Committee of the Regions  A Farm to Fork Strategy for a fair, healthy and environmentally-friendly food system.  COM/2020/381 final  <https://eur-lex.europa.eu/legal-content/EN/TXT/?uri=celex%3A52020DC0381> | Non-binding | *2.1. Ensuring sustainable food production*  The excess of nutrients (especially nitrogen and phosphorus) in the environment, stemming from excess use and the fact that not all nutrients used in agriculture are effectively absorbed by plants, is another major source of air, soil and water pollution and climate impacts. It has reduced biodiversity in rivers, lakes, wetlands and seas. The Commission will act to reduce nutrient losses by at least 50%, while ensuring that there is no deterioration in soil fertility. This will reduce the use of fertilisers by at least 20% by 2030. | 2030 |
| Pesticides | Communication from the commission to the european parliament, the council, the european economic and social committee and the committee of the regions  Pathway to a Healthy Planet for All  EU Action Plan: 'Towards Zero Pollution for Air, Water and Soil'  COM(2021) 400 final  <https://eur-lex.europa.eu/legal-content/EN/ALL/?uri=COM%3A2021%3A400%3AFIN> | Non-binding | *2.1.The zero pollution ambition*  The zero pollution targets for 2030 Under EU law, Green Deal ambitions and in synergy with other initiatives, by 2030 the EU should reduce: … 3. by 25% the EU ecosystems where air pollution threatens biodiversity; 4. by 50% nutrient losses, the use and risk of chemical pesticides, the use of the more hazardous ones, and the sale of antimicrobials for farmed animals and in aquaculture; | 2030 |
|  | Directive 2009/128/EC of the European Parliament and of the Council of 21 October 2009 establishing a framework for Community action to achieve the sustainable use of pesticides  (Version 26/7/2019)  <http://data.europa.eu/eli/dir/2009/128/2019-07-26> | Binding as to the result to be achieved (Article 288 TFEU) | *Article 4 National Action Plans*  1. Member States shall adopt National Action Plans to set up their quantitative objectives, targets, measures and timetables to reduce risks and impacts of pesticide use on human health and the environment and to encourage the development and introduction of integrated pest management and of alternative approaches or techniques in order to reduce dependency on the use of pesticides. These targets may cover different areas of concern, for example worker protection, protection of the environment, residues, use of specific techniques or use in specific crops. | 2030 |
|  | Communication from the Commission to the European Parliament, the Council, the European Economic and Social Committee and the Committee of the Regions  A Farm to Fork Strategy for a fair, healthy and environmentally-friendly food system.  COM/2020/381 final  <https://eur-lex.europa.eu/legal-content/EN/TXT/?uri=celex%3A52020DC0381> | Non-binding | *2.1. Ensuring sustainable food production*  .. The Commission will take additional action to reduce the overall use and risk of chemical pesticides by 50% and the use of more hazardous pesticides by 50% by 2030. | 2030 |
| Biodiversity | Regulation (EU) 2024/1991 of the European Parliament and of the Council of 24 June 2024 on nature restoration and amending Regulation (EU) 2022/869  [*http://data.europa.eu/eli/reg/2024/1991/oj*](http://data.europa.eu/eli/reg/2024/1991/oj) | Binding | *Article 1 Subject Matter*  2. This Regulation establishes a framework within which Member States shall put in place effective and area-based restoration measures with the aim to jointly cover, as a Union target, throughout the areas and ecosystems within the scope of this Regulation, at least 20 % of land areas and at least 20 % of sea areas by 2030, and all ecosystems in need of restoration by 2050. | 2030; 2050 |
|  | Regulation (EU) 2024/1991 of the European Parliament and of the Council of 24 June 2024 on nature restoration and amending Regulation (EU) 2022/869  [*http://data.europa.eu/eli/reg/2024/1991/oj*](http://data.europa.eu/eli/reg/2024/1991/oj) | Binding | *Article 4 Restoration of terrestrial, coastal and freshwater ecosystems*  1. Member States shall put in place the restoration measures that are necessary to improve to good condition areas of habitat types listed in Annex I which are not in good condition. Such restoration measures shall be put in place:   a) by 2030 on at least 30 % of the total area of all habitat types listed in Annex I that is not in good condition, as quantified in the national restoration plan referred to in Article 15 b) by 2040 on at least 60 % and by 2050, on at least 90 % of the area of each group of habitat types listed in Annex I that is not in good condition, as quantified in the national restoration plan referred to in Article 15.   10. The restoration measures referred to in paragraphs 1 and 4 shall consider the need for improved connectivity between the habitat types listed in Annex I and take into account the ecological requirements of the species referred to in paragraph 7 that occur in those habitat types. | 2030;  2050 |
|  | Regulation (EU) 2024/1991 of the European Parliament and of the Council of 24 June 2024 on nature restoration and amending Regulation (EU) 2022/869  [*http://data.europa.eu/eli/reg/2024/1991/oj*](http://data.europa.eu/eli/reg/2024/1991/oj) | Binding | *Article 12 Restoration of forest ecosystems*  1. Member States shall put in place the restoration measures necessary to enhance biodiversity of forest ecosystems, in addition to the areas that are subject to restoration measures pursuant to Article 4(1), (4) and (7), while taking into account the risks of forest fires.  2. Member States shall achieve an increasing trend at national level of the common forest bird index, as further specified in Annex VI, measured in the period from ... [the date of entry into force of this Regulation] until 31 December 2030, and every six years thereafter, until the satisfactory levels as set in accordance with Article 14(5) are reached.  3. Member States shall achieve an increasing trend at national level of at least six out of seven of the following indicators for forest ecosystems, as further specified in Annex VI, chosen on the basis of their ability to demonstrate the enhancement of biodiversity of forest ecosystems within the Member State concerned. The trend shall be measured in the period from ... [the date of entry into force of this Regulation] until 31 December 2030, and every six years thereafter, until the satisfactory levels as set in accordance with Article 14(5) are reached: (a) standing deadwood; (b) lying deadwood; (c) share of forests with uneven-aged structure (d) forest connectivity; (e) stock of organic carbon; (f) share of forests dominated by native tree species; (g) tree species diversity. | 2030+ |
|  | Regulation (EU) 2024/1991 of the European Parliament and of the Council of 24 June 2024 on nature restoration and amending Regulation (EU) 2022/869  [*http://data.europa.eu/eli/reg/2024/1991/oj*](http://data.europa.eu/eli/reg/2024/1991/oj) | Binding | *Article 9 Restoration of the natural connectivity of rivers and natural functions of the related floodplains*  1. Member States shall make an inventory of artificial barriers to the connectivity of surface waters and, taking into account the socio-economic functions of the artificial barriers, identify the barriers that need to be removed to contribute to meeting the restoration targets set out in Article 4 of this Regulation and fulfilling the objective of restoring at least 25 000 km of rivers into free-flowing rivers in the Union by 2030, without prejudice to Directive 2000/60/EC, in particular Article 4(3), (5) and (7) thereof, and Regulation (EU) No 1315/2013 of the European Parliament and of the Council46, in particular Article 15 thereof.  2. Member States shall remove the artificial barriers to the connectivity of surface waters identified in the inventory made pursuant to paragraph 1 of this Article, in accordance with the plan for their removal referred to in Article 15(3), points (i) and (n). When removing artificial barriers, Member States shall primarily address obsolete barriers, namely those that are no longer needed for renewable energy generation, inland navigation, water supply, flood protection or other uses. 46 Regulation (EU) No 1315/2013 of the European Parliament and of the Council of 11 December 2013 on Union guidelines for the development of the trans-European transport network and repealing Decision No 661/2010/EU (OJ L 348, 20.12.2013, p. 1)  3. Member States shall complement the removal of artificial barriers in accordance with paragraph 2 by the measures necessary to improve the natural functions of the related floodplains.  4. Member States shall ensure that the natural connectivity of rivers and natural functions of the related floodplains restored in accordance with paragraphs 2 and 3 are maintained. | 2030 |
|  | Regulation (EU) 2024/1991 of the European Parliament and of the Council of 24 June 2024 on nature restoration and amending Regulation (EU) 2022/869  [*http://data.europa.eu/eli/reg/2024/1991/oj*](http://data.europa.eu/eli/reg/2024/1991/oj) | Binding | *Article 8 Restoration of urban ecosystems*  1. By 31 December 2030, Member States shall ensure that there is no net loss in the total national area of urban green space and of urban tree canopy cover in urban ecosystem areas, determined in accordance with Article 14(4), compared to ... [year of entry into force of this Regulation]. For the purposes of this paragraph, Member States may exclude from those total national areas the urban ecosystem areas in which the share of urban green space in the urban centres and urban clusters exceeds 45 % and the share of urban tree canopy cover exceeds 10 %.  2. From 1 January 2031, Member States shall achieve an increasing trend in the total national area of urban green space, including through the integration of urban green space into buildings and infrastructure, in urban ecosystem areas, determined in accordance with Article 14(4), measured every six years from 1 January 2031, until a satisfactory level as set in accordance with Article 14(5) is reached  3. Member States shall achieve, in each urban ecosystem area, determined in accordance with Article 14(4), an increasing trend of urban tree canopy cover, measured every six years from 1 January 2031, until the satisfactory level identified as set in accordance with Article 14(5) is reached. | 2030;  2030+ |
|  | Regulation (EU) 2024/1991 of the European Parliament and of the Council of 24 June 2024 on nature restoration and amending Regulation (EU) 2022/869  [*http://data.europa.eu/eli/reg/2024/1991/oj*](http://data.europa.eu/eli/reg/2024/1991/oj) | Binding | *Article 11 Restoration of agricultural ecosystems*  1. Member States shall put in place the restoration measures necessary to enhance biodiversity in agricultural ecosystems, in addition to the areas that are subject to restoration measures under Article 4(1), (4) and (7), taking into account climate change, the social and economic needs of rural areas and the need to ensure sustainable agricultural production in the Union.  2. Member States shall put in place measures which shall aim to achieve an increasing trend at national level of at least two out of the three following indicators for agricultural ecosystems, as further specified in Annex IV, measured in the period from ... [the date of entry into force of this Regulation] until 31 December 2030, and every six years thereafter, until the satisfactory levels as set in accordance with Article 14(5) are reached: (a) grassland butterfly index; (b) stock of organic carbon in cropland mineral soils; (c) share of agricultural land with high-diversity landscape features.  3. Member States shall put in place restoration measures which shall aim to ensure that the common farmland bird index at national level based on the species specified in Annex V, indexed on ... [date: the first day of the month following 12 months from the date of entry into force of this Regulation] = 100, reaches the following levels: (a) for Member States listed in Annex V with historically more depleted populations of farmland birds: 110 by 2030, 120 by 2040 and 130 by 2050; (b) for Member States listed in Annex V with historically less depleted populations of farmland birds: 105 by 2030, 110 by 2040 and 115 by 2050.  4. Member States shall put in place measures which shall aim to restore organic soils in agricultural use constituting drained peatlands. Those measures shall be in place on at least: (a) 30 % of such areas by 2030, of which at least a quarter shall be rewetted; (b) 40 % of such areas by 2040, of which at least a third shall be rewetted; (c) 50 % of such areas by 2050, of which at least a third shall be rewetted. Member States may put in place restoration measures, including rewetting, in areas of peat extraction sites and count those areas as contributing to meeting the respective targets referred to in the first subparagraph, points (a), (b) and (c).  In addition, Member States may put in place restoration measures to rewet organic soils that constitute drained peatlands under land uses other than agricultural use and peat extraction and count those rewetted areas as contributing, up to a maximum of 40 %, to meeting the targets referred to in the first subparagraph, points (a), (b) and (c). Restoration measures that consist in rewetting peatland, including the water levels to be achieved, shall contribute to reducing greenhouse gas net emissions and increasing biodiversity, while taking national and local circumstances into account. | 2030;  2050 |
|  | Regulation (EU) 2024/1991 of the European Parliament and of the Council of 24 June 2024 on nature restoration and amending Regulation (EU) 2022/869  [*http://data.europa.eu/eli/reg/2024/1991/oj*](http://data.europa.eu/eli/reg/2024/1991/oj) | Binding | *Article 10 Restoration of pollinator populations*  1. Member States shall, by putting in place in a timely manner appropriate and effective measures, improve pollinator diversity and reverse the decline of pollinator populations at the latest by 2030 and thereafter achieve an increasing trend of pollinator populations, measured at least every six years from 2030, until satisfactory levels are achieved, as set in accordance with Article 14(5). | 2030;  2030+ |
|  | Directive 2009/147/EC of the European Parliament and of the Council of 30 November 2009 on the conservation of wild birds  (Version 26/06/2019)  <http://data.europa.eu/eli/dir/2009/147/2019-06-26> | Binding as to the result to be achieved (Article 288 TFEU) | *Article 2*  Member States shall take the requisite measures to preserve, maintain or reestablish a sufficient diversity and area of habitats for all the species of birds referred to in Article 1 |  |
|  | Council Directive 92/43/EEC of 21 May 1992 on the conservation of natural habitats and of wild fauna and flora  (Version 01/07/2013)  <http://data.europa.eu/eli/dir/1992/43/2013-07-01> | Binding as to the result to be achieved (Article 288 TFEU) | *Article 2*  1. The aim of this Directive shall be to contribute towards ensuring bio-diversity through the conservation of natural habitats and of wild fauna and flora in the European territory of the Member States to which the Treaty applies.  2. Measures taken pursuant to this Directive shall be designed to maintain or restore, at favourable conservation status, natural habitats and species of wild fauna and flora of Community interest.  3. Measures taken pursuant to this Directive shall take account of economic, social and cultural requirements and regional and local characteristics. |  |
|  | Regulation (EU) No 1143/2014 of the European Parliament and of the Council of 22 October 2014 on the prevention and management of the introduction and spread of invasive alien species  (Version 14/12/2019)  <http://data.europa.eu/eli/reg/2014/1143/2019-12-14> | Binding | *Article 7 Restrictions*  2. Member States shall take all necessary steps to prevent the unintentional introduction or spread, including, where applicable, by gross negligence, of invasive alien species of Union concern. |  |
|  | Communication from the commission to the european parliament, the council, the european economic and social committee and the committee of the regions  EU Biodiversity Strategy for 2030 Bringing nature back into our lives  COM(2020) 380 final  <https://eur-lex.europa.eu/legal-content/EN/TXT/HTML/?uri=CELEX:52020DC0380> | Non-binding | *2. Protecting and restoring nature in the European Union*  Nature protection: key commitments by 2030: 1.Legally protect a minimum of 30% of the EU’s land area and 30% of the EU’s sea area and integrate ecological corridors, as part of a true Trans-European Nature Network. 2.Strictly protect at least a third of the EU’s protected areas, including all remaining EU primary and old-growth forests. 3.Effectively manage all protected areas, defining clear conservation objectives and measures, and monitoring them appropriately.  EU Nature Restoration Plan: key commitments by 2030:  1. Legally binding EU nature restoration targets to be proposed in 2021, subject to an impact assessment. By 2030, significant areas of degraded and carbon-rich ecosystems are restored; habitats and species show no deterioration in conservation trends and status; and at least 30% reach favourable conservation status or at least show a positive trend. 2. The decline in pollinators is reversed. 3. The risk and use of chemical pesticides is reduced by 50% and the use of more hazardous pesticides is reduced by 50%. 4. At least 10% of agricultural area is under high-diversity landscape features. 5. At least 25% of agricultural land is under organic farming management, and the uptake of agro-ecological practices is significantly increased. 6. Three billion new trees are planted in the EU, in full respect of ecological principles. 7. Significant progress has been made in the remediation of contaminated soil sites. 8. At least 25,000 km of free-flowing rivers are restored. 9. There is a 50% reduction in the number of Red List species threatened by invasive alien species. 10. The losses of nutrients from fertilisers are reduced by 50%, resulting in the reduction of the use of fertilisers by at least 20%. 11. Cities with at least 20,000 inhabitants have an ambitious Urban Greening Plan. 12. No chemical pesticides are used in sensitive areas such as EU urban green areas. 13. The negative impacts on sensitive species and habitats, including on the seabed through fishing and extraction activities, are substantially reduced to achieve good environmental status. 14. The by-catch of species is eliminated or reduced to a level that allows species recovery and conservation. | 2030 |

Table S2: The full pathway narratives for each thematic cluster and objective, as well as the overall narrative for each NFF value perspective.

| **Pathway thematic cluster and objective** | **Nature For Nature** | **Nature As Culture** | **Nature For Society** |
| --- | --- | --- | --- |
| **Overall EU narratives** | The EU prioritises the strict protection of large areas where ecological processes are restored with minimal human management, placing emphasis on land sparing approaches.  With land being prioritised for nature, demand on land is met by land sparing approaches that sustainably intensify in high yield areas that are of least importance for biodiversity, while organic farming uses agro-ecological and plant breeding solutions to increase yields where possible. Renewable energy and land use policies are tailored to reduce ecological impact. Restoration practices with the most effective outcome for biodiversity are prioritised. | The EU places emphasis on land sharing and multifunctional landscapes which integrate nature into all aspects of local culture.  Agriculture uses traditional, nature-positive land management practices leading to an emphasis on local solutions, as opposed to widespread technological interventions. Renewable energy and land use policies are tailored to maintain the integrity of cultural landscapes. Restoration practices focus on restoring native and culturally important species in cultural landscapes. | The EU balances the supply of ecosystem services for society with nature in multifunctional landscapes.  Sustainable intensification of the most productive land utilises upscaled technology to increase the efficiency of inputs. Organic farming methods are used where possible to provide multiple ecosystem services. Renewable energy and land use policies incorporate technologies to increase the cost-effectiveness of integrating energy sources into multifunctional landscapes. Restoration practices focus on restoring the function of ecosystems to enhance the supply of ecosystem services. |
| **Organic food production:** At least 25% of the EU’s agricultural land under organic farming by 2030 | Farmland deemed as being most important for biodiversity (including High Nature Value Farmland) is prioritised for conversion to organic production. Yield increases from organic production are pursued with innovations in agro-ecological practices, promoting a land sparing approach. | To meet the organic food production ambition, conventional farmland deemed most culturally valuable is prioritised for conversion to organic production, such that organic food systems become well integrated into local society and culturally significant landscapes. | Conversion of farmland to organic production is prioritised in areas where it improves the supply of ecosystem services, for example groundwater quality. Organic land is allocated such that it avoids high-productivity areas to prevent conflicts with food security. |
| **Renewable energy:** a share of at least 42% of renewable energy consumption by 2030 | To increase the share of renewable energy consumption, there is a focus on solar and wind farms. The specific energy source is tailored to local conditions to prevent negative impacts on biodiversity, including spatial arrangements that maintain connectivity between natural areas. Large-scale installations are restricted to the least ecologically valuable locations. | Increased consumption of renewable energy is enabled through integrating renewable energy into multifunctional landscapes, such as through the use of small-scale agri-voltaics and widespread use of existing buildings for photovoltaic placement, instead of widespread solar and wind farms. Utility-scale energy production sites are located outside of landscapes valuable for nature and culture. | For the renewable energy target, renewable energy sources are selected on the basis of balancing cost-efficient energy production with the provision of other ecosystem services. In areas less important for provisioning other ecosystem services, high-intensity solar and wind farms are used, whilst multifunctional solutions, such as agri-voltatics, are used where beneficial for society. |
| **3 billion additional trees by 2030** | To meet the three billion additional trees pledge, a climate-resilient mix of trees are planted, with a preference for native species wherever possible. These are located on abandoned farmland, on previously intensively managed farmland or used in the regeneration of degraded forests in full respect of ecological principles to support ecosystem functioning. | To meet the 3 billion tree ambition, a selection of climate resilient, culturally significant species are planted in accessible areas, facilitating a greater connection between people and nature. Local communities are involved in planting the trees and protecting the reforested area. Some tree planting is included in the expansion of existing traditional agroforestry systems. Local perceptions of natural landscapes as open and semi-open habitats are, however, prioritised over tree planting where relevant. | To achieve the 3 billion additional trees pledge, climate resilient tree species are prioritised (regardless of their nativeness) and planted in areas that provide additional benefits to society, such as shade to reduce heat island effects, the reduction of air pollutants, or the improvement of soil retention on managed land. There is a significant focus on planting trees best for climate change mitigation (regardless of being native or exotic), to increase the offsetting possible in the carbon market. Agroforestry systems are implemented to provide multiple ecosystem service benefits and to reduce wildfire risk. |
| **Carbon**:  Pursuing to hold the increase in global average temperature to well below 2°C above pre-industrial levels, society aims to reduce GHG emission by 55% compared to 1990 levels in 2030, and reach net zero emissions by 2050. | Multiple land strategies are used to reduce greenhouse gas emissions. Society meets its energy requirements with the upscaling of wind and solar energy sources that utilise technology to reduce impacts on biodiversity. Larger renewable installations are restricted to the least ecologically valuable locations. Emissions reductions from the land sector are secondary to meeting biodiversity targets, with faster emission reductions in other sectors limiting the need for a compensating LULUCF sink. Current carbon sinks are protected by avoiding deforestation of ecologically functional and old growth forests. Increases in the carbon sink focus on restoring heterogenous forest landscapes with a mix of dense, light and open landscapes using extensive forest management along with spontaneous tree regeneration. Peatlands and other wetlands are restored for biodiversity, with potential synergies in emission reductions. No agriculture is allowed on (restored) peatlands or other wetlands. | To meet the reduction in greenhouse gases, society meets its energy requirements with the upscaling of renewable and bioenergy sources that minimally alter cultural landscapes. Larger renewable installations are restricted to areas where they don't interfere with humans. Reducing emissions in the land sector is secondary to maintaining cultural landscapes, with faster emission reductions in other sectors limiting the need for a compensating LULUCF sink. Nature-based solutions (e.g. the use of wood as construction material) are promoted unless they interfere with local culture. Increases in carbon sinks are achieved by avoiding deforestation and promoting forest management with selective logging for long-lived wood products and a focus on planting native and culturally important tree species. Peatland and other wetland restoration prioritises cultural benefits alongside emissions reductions. Extensive forms of agriculture (e.g. paludiculture) are allowed on restored peatlands and other wetlands, as well as rewetting peatlands allowing experiences and activities that foster human-nature interactions. | To reduce greenhouse gas emissions, society meets its energy requirements with the upscaling of renewable energy sources, including both large (solar and wind farms) and small-scale renewable installations (agri-voltaics). Bioenergy production focuses on small-scale 2nd generation energy crops and residual forest products. Emissions reductions from the land sector occur via promoting a variety of nature-based solutions that provide benefits for biodiversity and people. Increases in carbon sinks are achieved by avoiding deforestation, and managing the forests through selective logging (allowed to facilitate the production of long-lived wood products), and planting tree species that provide long-term climate benefits. Peatlands and other wetland restoration permits paludiculture and rewetting for recreational purposes that foster economic benefits for local communities. |
| **Water quality and quantity:** Prevent deterioration and improve the status of bodies of surface water and groundwater, Reduce concentration of pollutants in groundwater. | Improvements in water quality and quantity come from a ban on water abstraction for irrigation in ecologically sensitive areas, and only minimal use of nitrogen fertilisers is permitted to minimise impacts on biodiversity. In more intensive agricultural areas, nitrogen fertiliser use is managed using precision technologies to reduce runoff. Water bodies are restored to achieve benefits for biodiversity. | Only minimum and targeted use of fertilisers is allowed, with a focus on traditional fertilisation and manuring practices. Water body restoration is balanced with cultural ecosystem services that are unsubstitutable to people. | To improve water quality and quantity, fertiliser application is allowed in areas of high agricultural management intensity, while aiming to balance water quality and food production on a landscape-scale. A reduction of fertiliser is aligned to prevent significant yield losses, by using technology such as precision agriculture and vertical farming, as well as crop rotations with nitrogen-fixing species. Water bodies and wet- and peatlands are restored to supply provisioning and regulating ecosystem services, such as reducing the risk of flooding. |
| **Pesticides**:  By 2030, the EU should reduce by 50% nutrient losses, the use and risk of chemical pesticides, the use of the more hazardous ones, and the sale of antimicrobials for farmed animals and in aquaculture | Pesticides are totally removed from use in key biodiversity areas. There is targeted use of pesticides in sustainably managed intensive agricultural landscapes, to minimise usage and seepage into the environment. | Reductions in fertiliser usage comes through allowing only minimum and targeted use, with a focus on traditional fertilisation and manuring practices. Water body restoration is balanced with cultural activities and people’s non-material connection to water. Pesticides are reduced in cultural landscapes, especially where they threaten culturally important species, such as bees for beekeeping. | Pesticides are also allowed in moderation, with the goal to balance water quality and food production, using similar technologies. |
| **Biodiversity - restoration**:  By 2050, 90% of habitat areas in need of restoration should be improved to good condition. There is improved connectivity between habitat types and area-based restoration measures for all ecosystems where needed. | The focus of restoration efforts is on creating the conditions such that functioning ecosystems can evolve. This is enabled by restoring large, geodiverse single and cumulative natural and semi-natural areas with high trophic complexity, effective dispersal processes within and between them, and natural disturbance regimes. Habitat connectivity is improved for habitats with high biodiversity value. All types of landscape modification that improve connectivity are allowed, including connecting low and high biodiversity areas to provide additional refuge. Habitat restoration focuses on restoring natural stochastic disturbance regimes and all trophic levels of the ecosystems. | The focus of restoration is on habitats of species in traditionally important landscapes, such as heathlands or alpine grasslands. Restoration efforts also include the maintenance and introduction of traditional management practices to keep cultural landscapes. Habitat connectivity is improved for species in cultural landscapes and habitat restoration focuses on trophic complexity and emblematic endemic species that do not conflict with traditional land use practices and landscape features such as hedgerows, ponds and stone walls. | The focus of restoration is on habitats and species that provide more ecosystem services. Habitat connectivity is improved for species in agricultural and rural landscapes, while not impairing material benefits. Habitat restoration focuses on climate-smart solutions, specifically on carbon storage and co-benefits with other regulating ecosystem services, as well as actions to reduce the risks to lives and assets from extreme weather events. |
| **Biodiversity - forests**: There is an increasing trend in the common forest bird and in 6 out of 7 of the following: standing deadwood, lying deadwood, the share of forests with uneven age, connectivity, organic carbon, share of forests dominated by native tree species and tree species diversity. | In native or ecologically equivalent forests, a diverse, ecosystem-appropriate mix of species are promoted in standing trees, deadwood, and open and semi-open areas within woodlands. No extractive use of ecologically functional forests is allowed, whilst limited extractive use is permitted where transitionary management and forest restructuring support climate resilience. Ecosystem restoration to enhance connectivity focuses on improving habitat for native animal species, whilst simultaneously increasing the stock of organic carbon. | In forests, small amounts of timber extraction is allowed for construction of sustainable, traditional housing, using mixed-age timber to increase the unevenness of the forest age and to allow for the build-up of deadwood and organic carbon. Small forest patches and other woody features are protected as landscape features in agricultural ecosystems. | In forests, management focuses on increasing carbon stock for benefits to climate change mitigation, by increasing standing deadwood and reducing fire risk. Extractive use of forest resources is permitted in a more sustainable way to increase the unevenness of the forest age and soil organic carbon to promote more adapted resilient forests. |
| **Biodiversity - rivers**: Restore at least 25000 km of rivers into free-flowing rivers by 2030 | River ecosystems are restored by removing anthropogenic barriers to rivers, such as hydrological dams, weirs and locks, where possible, to allow for more natural river dynamics. Rivers are allowed to change course over time and reconnect to floodplains. Additionally, ecosystem engineers of rivers, such as beavers and water voles, are reintroduced. | Restoration of river systems includes removal of river barriers in landscapes that allow for cultural practices, fostering experiences and spiritual connections with nature. Local communities participate in the watershed management. | In the freshwater system, flood risk is reduced through river dredging and partially diverting rivers around urban settlements through newly meandering routes, whilst simultaneously allowing for additional access points for recreation in rivers. Natural retention basins are created to reduce flooding downstream, such as by setting aside meadows that are allowed to flood. |
| **Biodiversity - urban areas**:  Increase the total national area and urban tree canopy cover in urban ecosystems. | Urban green spaces maintain native biodiversity while allowing access to people for recreational use. Connections to natural habitats are developed via nature corridors, where native flora and fauna is introduced and managed according to biodiversity needs. Multi-species habitat-mosaics are promoted in formerly open green spaces. Vegetated areas take priority over new developments, allowing a multi-species habitat mosaic to flourish. This includes tree health and abundance as well as spontaneous vegetations. Living walls and rooftop gardens are created and valued for the increase in biodiversity they bring to urban areas. | Urban green spaces are actively managed by the local area to enable further experiences and spiritual connections with nature. New green spaces are created through initiatives such as living walls and rooftop gardens, and facilitate greater everyday interaction with nature in urban areas. Green spaces are mixed, consisting of both forested and open greenspaces. Additional green spaces are included in areas with less access to natural green spaces, such as on disused building sites and brownfield sites, to facilitate greater integration of nature as part of the urban culture. Society is engaged in the greening of their gardens with a focus on traditional species. Community gardens provide an important opportunity to share traditional sustainable farming practices in an urban setting. | Urban green spaces are focused on provisioning and cultural ecosystem services, for example through urban gardening for food production and by increasing trees to help with climate regulation. Living walls are incorporated into buildings and rooftops are used for additional green space, to allow for increased building developments as well as increased green space. |
| **Biodiversity - agricultural landscapes**: Restore 50% of organic soils in areas of drained peatlands by 2050, with at least a third rewetted. By 2030, there should be an increasing trend of the common farmland bird index and of at least 2 of 3 of: the grassland butterfly index, the stock of organic carbon, land with high-diversity landscape features. | Restoration of agricultural ecosystems focuses on improving habitats that increase the grassland butterfly index and the farmlands bird index. To increase bird species and to create wildlife corridors, organic farms are spatially arranged within a matrix to promote connectivity and reduce ecosystem fragmentation. Food crops are highly diverse and locally adapted. Extensive pastures transition to trophic rewilding areas by introducing free-roaming herbivores to support threatened open and semi-open landscape species and prevent homogenous succession of this habitat to shrubland or forest, instead promoting development of mosaic vegetation. . | In agricultural ecosystems, restoration within cultural landscapes focuses on increasing the share of agricultural land with high-diversity landscape features. Additionally, the bird species that are prioritised to increase the overall farmland bird index are those with stronger cultural connections to nature or are spiritually important. Farmland bird populations are hence restored in high nature value farmland. | In agricultural ecosystems, restoration focuses on improvements in the functioning of systems that contribute to food production, such as through reintroduction of small landscape features in intensively farmed land or increasing the stock of organic carbon in cropland mineral soils. The farmland bird index rises due to the protection of birds that provide benefits to society, e.g., for pest control and recreational activities. |
| **Biodiversity - pollinators**: Reverse the decline of pollinator populations by 2030. | Measures are taken to increase population trends of all pollinator species, including land sparing and sustainable farming practices aimed at increasing habitat connectivity and quality for wild pollinators. | The increase in pollinators comes from culturally significant species, including those embedded in local traditions like beekeeping. This happens through an increase in pollinator-friendly plant species and a reduction of pesticides where possible, whilst maintaining traditional cultural landscapes. | Increases in pollinator diversity provides a pollination service to farmland, through improved pesticide management and improving floral and nesting resources. |
| **Biodiversity - invasive alien species**: Prevent the introduction of, control or eradicate those alien species which threaten ecosystems, habitats or species. Reduce by 50% the number of Red List species threatened by invasive alien species by 2030. | Suppression of invasive alien species focuses on those that threaten native species or ecosystem functioning. | The eradication of invasive alien species focuses on those that threaten native, culturally valued species, or those species needed for a functioning ecosystem. | The eradication of invasive alien species prioritises those that threaten the provision of important ecosystem services through their negative effects on ecosystem functioning. |
| **Biodiversity - Protected Areas**: By 2030, legally protect a minimum of 30% of the EU's land area and integrate ecological corridors. Strictly protect at least a third of this land. | The majority of the EU’s protected areas (PAs) are under strict protection according to IUCN categories ‘strict nature reserve’(*Ia*) or ‘wilderness areas’(*Ib*), with the aim to preserve the long-term integrity of these natural areas at an ecosystem scale and without disturbance from significant human activity. PAs are placed in locations which have the greatest ecological value, including all remaining old growth forests and other currently existing natural areas. To build a PA network, additional sites are restored to natural systems and protected to act as nature corridors, including the conversion of currently existing PAs in semi-natural and cultural landscapes to IUCN categories ‘wilderness areas’(*Ib*), ‘national park’(*II*) or ‘habitat or species management area’(*IV)*. A small proportion of PAs allows for limited and sustainable resource use, such as when subsistence resource use of local communities contributes to conserving a specific ecosystem where species have become dependent on human-modified landscapes. | The majority of Europe’s PAs are protected according to the IUCN category ‘protected landscape’ (*V*) and located in cultural landscapes and mainly co-managed with local communities, with the aim to protect biodiversity and conserve societies’ relational values of people with nature. This includes traditional management practices in agriculture and forestry that shape the landscape, or activities that foster meaningful and spiritual experiences in nature. A third of PAs are strictly protected, whereby PAs according to IUCN categories *Ia*, *Ib* and *II* are preferentially located in areas that do not require the conversion of human-shaped, culturally important landscapes. Stricter protection of specific local sites according to the IUCN category ‘natural monument or feature’ (*III*) occurs throughout cultural landscapes, in order to protect outstanding natural features with their associated biodiversity and to conserve traditional spiritual and cultural values of such sites. People are committed to nature conservation so the number of private protected areas increases. To build a PA network, additional sites are protected throughout and between cultural landscapes according to IUCN category *IV*, with the aim to provide managed nature corridors for key species. | The majority of PAs are under protection according to the IUCN category ‘protected area with sustainable use of natural resources’(*VI)*, with the aim to protect the supply of provisioning and regulating ecosystem services by means of sustainable use of natural resources. A third of PAs are strictly protected and located in areas where the benefits to regulating services outweigh the costs of prohibited resource use. Larger strictly protected areas are categorised as ‘national park’ (*II*) and still provide opportunities for tourism and recreation. Additionally, stricter protection of specific local sites according to the category ‘natural monument or feature’ (*III*) occurs throughout multifunctional landscapes, in order to protect outstanding natural features with their associated biodiversity for touristic and recreational purposes. To build a PA network, additional sites are protected throughout multifunctional landscapes according to category ‘habitat or species management area’ (*IV*), with the aim to provide managed nature corridors for key species for ecosystem functioning and society. |
